# Evaluation of Brain Microstructural Alterations in Preschool Autism Spectrum Disorder: A Voxel‐Wise Multimodal MRI Study

**DOI:** 10.1002/jmri.70185

**Published:** 2026-01-16

**Authors:** Changhao Wang, Meiying Cheng, Yu Lu, Jinxia Guo, Xueyan Liu, Zhanqi Feng, Shipeng Liu, Xin Zhao

**Affiliations:** ^1^ Department of Radiology the Third Affiliated Hospital of Zhengzhou University Zhengzhou China; ^2^ Henan International Joint Laboratory of Neuroimaging Zhengzhou China; ^3^ MR Research China, GE Healthcare Beijing China

**Keywords:** autism spectrum disorder, magnetic resonance imaging, preschool children, quantitative susceptibility mapping, T1 mapping

## Abstract

**Background:**

Autism Spectrum Disorder (ASD) presents with early neurodevelopmental alterations in preschool children, yet comprehensive characterization using multimodal quantitative MRI remains limited in this age group.

**Purpose:**

To investigate voxel‐wise brain microstructural differences in preschool ASD through integrated analysis of cerebral perfusion, multiparametric relaxometry, and magnetic susceptibility.

**Study Type:**

Prospective case–control.

**Population:**

Twenty nine‐children with ASD (age 2–6 years; 23 males/6 females) and 25 age‐/sex‐matched healthy controls (HC).

**Field Strength/Sequence:**

3.0 T MRI; high‐resolution 3D‐T1WI, quantitative susceptibility mapping (QSM), synthetic MRI (SyMRI), 3D pseudo‐continuous arterial spin labeling (3D‐pCASL).

**Assessment:**

Clinical assessments included the Gesell Developmental Schedules (GDS) and Childhood Autism Rating Scale (CARS). Imaging analysis consisted of voxel‐wise whole‐brain assessment of QSM, T1/T2/PD, and cerebral blood flow (CBF) maps.

**Statistical Tests:**

General linear models with cluster‐based thresholding were applied for group comparison; Spearman's rank correlation with Bonferroni correction was used for clinical associations; and receiver operating characteristic (ROC) analysis with Delong's test was performed to compare diagnostic performance based on the areas under the curve (AUCs).

**Results:**

Compared to HC, children with ASD showed decreased QSM values in the left superior/middle frontal gyri (SFG/MFG; cluster = 212 voxels, peak *T* = 5.55, *p* < 0.001). They also had reduced T1 relaxation times in bilateral SFG/MFG/precentral/postcentral gyri (four clusters: 315–750 voxels, peak *T* = 5.11–5.88, all *p* < 0.001). QSM values in the left SFG/MFG correlated positively with fine motor scores (*r* = 0.630, *p* < 0.001), while T1 values in the bilateral precentral/postcentral gyri correlated with gross motor scores (right: *r* = 0.548, *p* = 0.002; left: *r* = 0.461, *p* = 0.012). ROC analysis showed high diagnostic accuracy for both QSM (left SFG/MFG AUC = 0.858) and T1 values (left SFG/MFG AUC = 0.905; bilateral precentral/postcentral gyri AUC = 0.892–0.908).

**Data Conclusion:**

Preschool ASD demonstrates prefrontal iron deficiency (reduced QSM) and sensorimotor myelination alterations (decreased T1), which correlate with motor deficits and show high diagnostic efficacy.

**Evidence Level:**

2.

**Technical Efficacy:**

Stage 2.

## Plain Language Summary

- This study used advanced, non‐invasive MRI scans to compare the brains of 29 preschool children with autism spectrum disorder (ASD) and 25 typically developing children.
- The results showed that children with ASD had two key differences: reduced iron content in the prefrontal cortex and altered tissue structure (suggesting differences in myelination) in sensorimotor areas.
- Importantly, these brain changes were correlated with the children's scores on tests of fine and gross motor skills.
- These findings indicate that multimodal MRI can detect sensitive biomarkers of ASD, supporting its potential role in aiding early diagnosis and objective assessment.

## 1 Introduction

Autism spectrum disorder (ASD) is one of the most prevalent neurodevelopmental condition characterized by persistent deficits in social interaction and communication, alongside repetitive stereotyped behaviors and restricted interests [1]. According to data released by the World Health Organization, approximately one in 100 children worldwide is affected by ASD [2]. Currently, the diagnosis of ASD primarily relies on clinical evaluation based on the Diagnostic and Statistical Manual of Mental Disorders, Fifth Edition (DSM‐V), supplemented by standardized behavioral rating scales [3]. While these tools are effective for identifying core symptoms, they are subject to clinician interpretation and often depend on detailed developmental history provided by caregivers. This subjectivity can limit the consistency and early detection of ASD. Therefore, exploring objective diagnostic indicators, particularly neuroimaging biomarkers, may have important implications for early diagnosis, prognosis assessment, and individualized intervention strategies.

To date, no curative treatment exists for ASD. The mainstay of management involves behavioral and developmental rehabilitation, with the optimal window for intervention occurring before the age of six (6). Early identification, diagnosis and intervention have been shown to significantly improve developmental outcomes in children with ASD [4].

Traditional structural magnetic resonance imaging (MRI) often fails to detect visible abnormalities in children with ASD. However, an increasing body of evidence suggests that microstructural alterations in the brain are present [5, 6, 7]. For example, a voxel‐based morphometry study has reported volumetric changes in the middle temporal gyrus, superior temporal gyrus, postcentral gyrus (PostCG) and parahippocampal gyrus in adult patients with ASD [8]. Moreover, abnormalities in cortical thickness of patients with ASD, particularly in the frontal and temporal lobes have also been documented across multiple studies [9, 10]. These findings offer critical neuroanatomical insights into the pathophysiological mechanism underlying ASD.

Emerging quantitative MRI techniques, such as quantitative susceptibility mapping (QSM) and synthetic MRI (SyMRI), offer new opportunities to characterize subtle brain tissue changes. QSM noninvasively quantifies brain magnetic susceptibility, primarily reflecting iron and myelin content [11, 12]. Altered iron distribution, particularly in brain regions like the basal ganglia, has been reported in children with ASD [13, 14, 15]. SyMRI provides T1, T2 and PD maps, and enables estimation of myelin content, offering insight into neurodevelopment status [16, 17]. Arterial spin labeling (ASL) noninvasively measures cerebral blood flow (CBF), another marker of brain maturation. A study has revealed altered CBF patterns in children with ASD, particularly in the left frontal lobe, bilateral parietal lobes, and bilateral temporal lobes [18]. Furthermore, longitudinal evidence demonstrates a progressive expansion of hypoperfused regions with advancing age in the autistic population [19].

While many studies have independently investigated iron deposition, myelination, or perfusion in children with ASD few have examined concordant alterations across multiple quantitative MRI biomarkers [20, 21, 22]. Such co‐occurring changes may reflect shared pathophysiological mechanisms, such as oxidative stress or inflammation, that underlie both ASD and its common comorbidities. This study aimed to integrate QSM, SyMRI, and ASL to assess brain microstructural alterations in young children with ASD, examine associations between co‐localized biomarkers and developmental or symptom severity scores, and evaluate their potential value in early diagnostic assessment.

## 2 Materials and Methods

### 2.1 Participants

**FIGURE 1:**
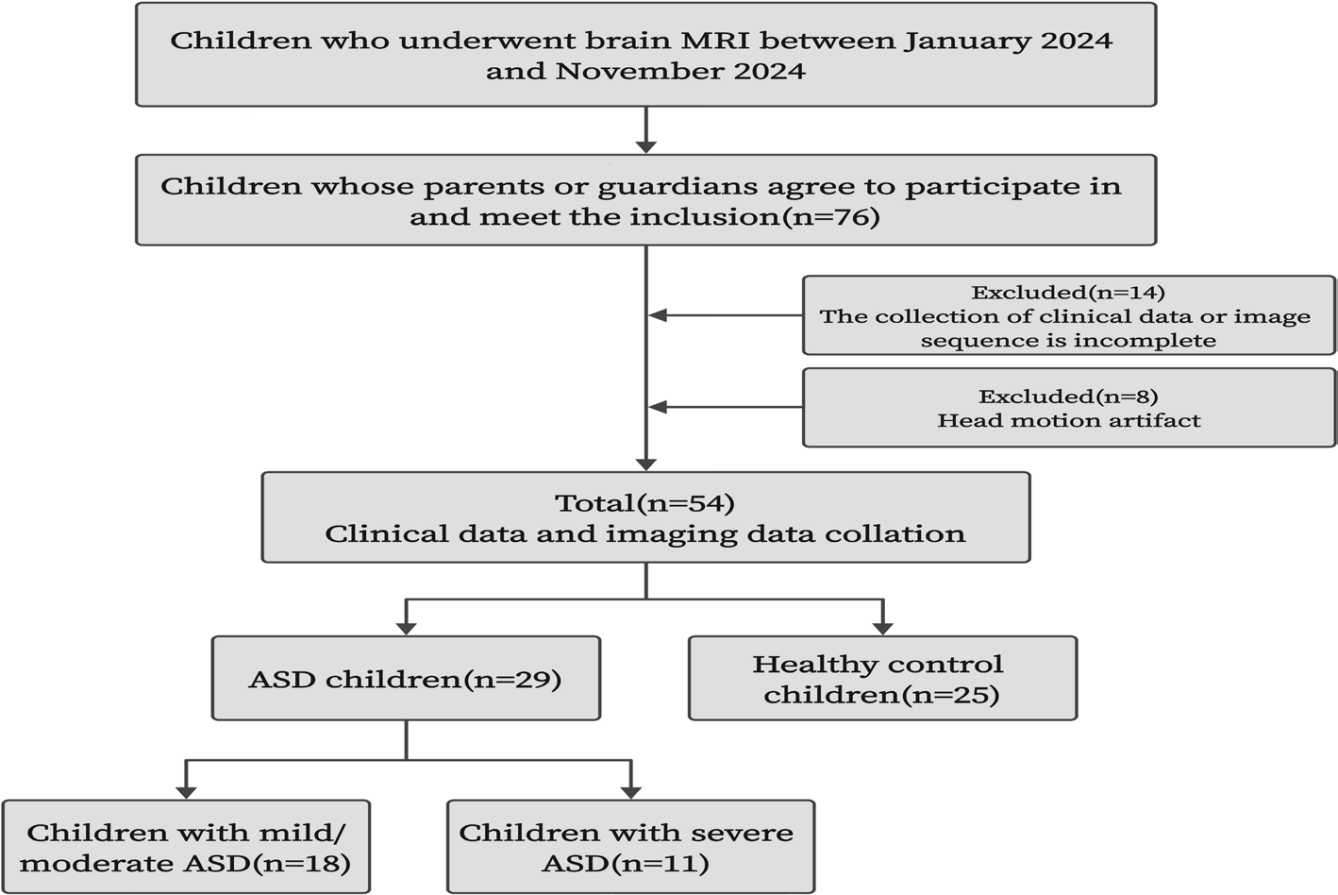
Flow chart of participant inclusion and exclusion.

Children who were newly diagnosed with ASD in the Children's Developmental Behavior Center of the Third Affiliated Hospital of Zhengzhou University between January 2024 and November 2024 were recruited, along with age‐and sex‐matched healthy control (HC) children. This study was approved by the Medical Ethics Committee of our hospital (No. 2024‐105‐01). Detailed medical histories were obtained from all participants prior to MRI examination, and written informed consent was obtained from the guardians of all participants. The inclusion and exclusion criteria for the ASD and HC groups are summarized in Table 1. A detailed flowchart of the participant screening, inclusion, and exclusion steps is presented in Figure 1.

**TABLE 1:** Inclusion and Exclusion Criteria for the ASD and Healthy Control Groups.

Criteria	ASD group	Healthy control group
Inclusion	Diagnosis of ASD based on DSM‐5 criteria Age 2–6 years at the time of diagnosis Full‐term birth (≥ 37 weeks) Right‐handed, Han nationality, native Chinese speaker No prior drug or psychological treatment	Age 2–6 years Full‐term birth (≥ 37 weeks) Right‐handed, Han nationality, native Chinese speaker Normal development in social, motor, and language development Normal brain MRI No history of neurological or psychiatric disorders Normal blood test results – Serum iron: 6.5–9.85 μmol/L – Hemoglobin (HGB): 110–150 g/L – Hematocrit (HCT): 35%–50% – Mean corpuscular volume (MCV): 80–100 fL
Exclusion	History of neurological disorders, head trauma, family history of neurological disorders, or psychotropic medication use Preterm birth (< 37 weeks) or significant birth defects Abnormal brain MRI findings MRI contraindications Incomplete clinical data or missing imaging sequences Presence of significant head motion artifacts on MRI	

### 2.2 Clinical and Neuropsychological Assessments

All participants were assessed and diagnosed by experienced clinicians (with > 10 years of experience) at our hospital. Children in the ASD group underwent comprehensive clinical evaluations using standardized neurodevelopmental and behavioral rating scales, including the Gesell Developmental Schedules (GDS) [23] and the Childhood Autism Rating Scale (CARS) [24]. Additionally, peripheral blood samples were collected from all participants to measure trace element iron content, hemoglobin (HGB), hematocrit (HCT), and mean corpuscular volume (MCV).

The GDS assesses five developmental domains: adaptive behavior (DQ1), gross motor skills (DQ2), fine motor skills (DQ3), language (DQ4) and personal social behavior (DQ5). The development quotient (DQ), which is the average of DQ1 through DQ5, categorizes neurodevelopmental status into six levels: (1) DQ > 86, normal development; (2) 76 ≤ DQ ≤ 85, borderline; (3) 55 ≤ DQ ≤ 75, mild developmental delay; (4) 40 ≤ DQ ≤ 54, moderate developmental delay; (5) 25 ≤ DQ ≤ 39, severe developmental delay; (6) DQ < 25, profound developmental delay.

The CARS evaluates autism severity across 15 behavioral items, with total scores classifying individuals as non‐autistic (total score < 30), mildly to moderately autistic (total score 30–36), or severely autistic (total score ≥ 36). Higher scores indicate more severe autism‐related symptoms.

### 2.3 Image Acquisition

All MRI scans were performed on a 3.0 T scanner (SIGNA Pioneer, GE Healthcare) using a 16‐channel combined head and neck coil. Prior to scanning, all participants received intravenous sedation with dexmedetomidine hydrochloride at a dose of 0.8 μg/kg. During image acquisition, participants wore rubber earplugs to reduce acoustic noise and were kept with blankets to maintain body temperature.

The sequences used for voxel‐wise analysis include high‐resolution three‐dimensional T1‐weighted imaging (HR 3D‐T1WI), QSM, synthetic magnetic resonance imaging (SyMRI) using the magnetic resonance image compilation (MAGiC) technique, and three‐dimensional pseudo‐continuous ASL (3D‐pCASL). Detailed scanning parameters for these core sequences are provided in Table 2. For 3D‐pCASL, labeling duration = 1500 ms, post‐labeling delay = 1525 ms, labeling slab thickness/gap = 3.35 mm/2 cm, and number of excitations (NEX) = 3.

**TABLE 2:** MRI acquisition parameters.

Parameter	TE/TR (ms)	FOV (mm^2^)	Thickness/spacing (mm)	Slices	FA (°)	Voxel size (mm^3^)	Matrix size	Acceleration type/Factor	Inversion times (ms)	Time
3D‐T_1_WI (Sag)	2.868/7.324	220 × 220	1/1	156	8	0.8594 × 0.8594 × 1.0	256 × 256	ASSET/2	600	2 min 40 s
QSM	14.616/25.664	240 × 240	2/2	1152	20	0.9375 × 0.9375 × 2.0	256 × 256	ASSET/2	—	2 min 20 s
SyMRI	17.072/5498	240 × 240	4/4	560	90	0.9375 × 0.9375 × 4.0	256 × 256	ASSET/2	12.028	5 min 00 s
3D‐pCASL	10.864/4650	240 × 240	4/4	72	111	1.875 × 1.875 × 4.0	512 × 512	SPIRAL_GEMS	1525	4 min 10 s

Abbreviations: 3D‐pCASL: 3D pseudo‐continuous arterial spin labeling; DWI: diffusion‐weighted imaging; FA: Flip Angle; FLAIR: fluid‐attenuated inversion recovery; FOV: field of view; QSM: quantitative susceptibility mapping; Sag: sagittal; SyMRI: synthetic magnetic resonance imaging; TE: echo time; TR: repetition time.

### 2.4 Data Preprocessing

QSM DICOM images were imported into STISuite v3.0 (https://people.eecs.berkeley.edu/~chunlei.liu/software.html) for whole brain susceptibility quantification. The processing pipeline included phase unwrapping, background field removal, and susceptibility inversion. Background field removal was performed using the V‐SHARP algorithm, which is less susceptible to erosion at the brain cortex compared to other methods, thus helping to preserve signal at the gray matter (GM) boundaries.

Data acquired with the MAGiC sequence were processed using SyMRI version 8.0 (SyntheticMR, Linköping, Sweden) to generate quantitative T1, T2, and proton density (PD) maps. Simultaneously, the software performed automated tissue segmentation, yielding volumetric measurements of GM, white matter (WM), myelin, and cerebrospinal fluid (CSF).

The 3D‐pCASL data were processed on an ADW 4.7 workstation (GE Healthcare) to generate CBF maps.

**FIGURE 2:**
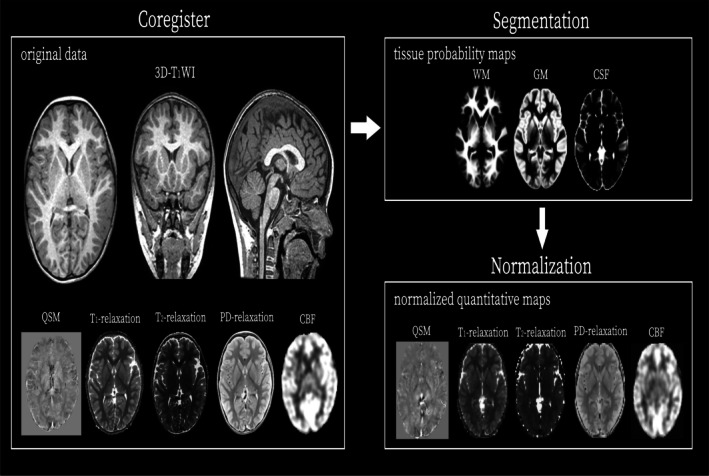
Schematic of the image processing workflow.

The HR 3D T1WI images were segmented into GM, WM and CSF images using the Chinese Pediatric Developmental Cohort (CHN‐PD) tissue probability maps (TPMs) (https://www.nitrc.org/projects/chn‐pd). These images were then spatially normalized to the Montreal Neurological Institute (MNI) standard space (http://www.mni.mcgill.ca/). All QSM, T1, T2, PD and CBF maps were subsequently co‐registered to the corresponding HR 3D‐T1WI and transformed to the MNI space using the same deformation fields. Finally, all spatially normalized images were smoothed using an 8 mm full width at half maximum (FWHM) isotropic Gaussian kernel. All image processing was conducted using Statistical Parametric Mapping version 12 (SPM12; Wellcome Centre for Human Neuroimaging, London, UK; http://www.fil.ion.ucl.ac.uk/spm/software/spm12) implemented in MATLAB (MathWorks, Natick, MA, USA) (Figure 2).

### 2.5 Statistical Analysis

Group‐level comparisons of clinical measures, GM, WM, and CSF volumes, as well as GSD and CARS scores were performed using R software (https://www.r‐project.org/). The Shapiro–Wilk test was used to evaluate the normality of continuous variables, while Levene's test was used to evaluate homogeneity of variance. Variables satisfying both assumptions were expressed as mean ± standard deviation ($\bar{x}$ *± s*) and compared using independent‐samples *t*‐tests. Variables violating either assumption were analyzed using the Wilcoxon rank‐sum test (Mann–Whitney *U* test) and reported as median [interquartile range, IQR; *P25*, *P75*]. Categorical variables were compared using the Chi‐square test.

Voxel‐wise comparisons were conducted between the ASD group and the HC group, as well as between the mild‐to‐moderate ASD subgroup and the severe ASD subgroup using general linear models (GLMs) for QSM, T1, T2, PD, and CBF maps. Age, sex, and intracranial volume (ICV) were included as covariates.

Statistical significance was defined using a voxel‐level height threshold of *p* < 0.001 (uncorrected), combined with a cluster‐level threshold of *p* < 0.05 corrected for family‐wise error (FWE). In addition, only clusters exceeding 100 contiguous voxels were considered in the final results.

In addition to the voxel‐wise analyses performed for all quantitative maps (QSM, T1, T2, PD, and CBF), an exploratory region‐of‐interest (ROI) analysis was conducted to further evaluate potential group differences in iron‐related regions. Specifically, the basal ganglia were selected given their known relevance to iron deposition and previously reported alterations in ASD. Using the AAL3 atlas, mean QSM values were extracted from the bilateral caudate nucleus, putamen, pallidum, and thalamus from the unsmoothed, normalized QSM maps for each participant. Group differences were assessed using independent‐sample *t*‐tests, and the results were FDR‐corrected for multiple comparisons.

To further examine associations between aberrant brain regions and developmental or symptom severity in children with ASD, clusters showing significant between‐group differences were defined as ROIs. For each participant, the mean quantitative value within each ROI was extracted and Spearman's rank‐order correlation analyses were performed with the GDS and the CARS scores. The significance threshold for these correlation analyses was adjusted using the Bonferroni correction (*p* = 0.05 divided by the number of significant ROIs).

To assess the diagnostic value, receiver operating characteristic (ROC) curve analyses were conducted using mean ROI values from clusters showing significant voxel‐wise group differences. Area under the curve (AUC) and its 95% confidence interval (CI) were calculated to evaluate the discriminatory performance. Comparisons between the AUCs from the multi‐modal approach (QSM and T1 values) and those from individual QSM or T1 parameters were performed using DeLong's test in R.

## 3 Results

### 3.1 Demographic Data

In total, 76 children met the inclusion criteria and had parental or guardian consent to participate (Figure 1). Of these, 10 children with ASD and 12 HC children were excluded due to unexpected arousal sedation leading to head motion artifacts, incomplete clinical data, or missing imaging sequences. Ultimately, 29 children with ASD and 25 HCs, aged between 2 and 6 years, were included in the study. There were no significant differences in age, sex, or other clinical parameters between the ASD and control groups (*p* > 0.05; Table 3). Additionally, when stratified by ASD severity, no significant differences were observed in demographic or clinical variables between the mild‐to‐moderate and severe ASD groups (*p* > 0.05; Table 4).

**TABLE 3:** Comparison of characteristics and assessment scores between the ASD and HC groups.

	ASD (*N* = 29)	HC (*N* = 25)	*p*
Gender (male/female)	23/6	20/5	0.950
Age (months)	48.00 (36.00,48.00)	36.00 (36.00, 48.00)	0.254
Weight (kg)	16.17 ± 5.73	16.27 ± 3.61	0.973
SyMRI			
ICV (mL)	1358.83 ± 139.98	1311.04 ± 204.43	0.330
WM (mL)	374.00 (327.60, 392.70)	332.20 (317.00, 356.10)	0.067
GM (mL)	694.30 (591.90, 750.30)	686.80 (629.50, 722.30)	> 0.999
MY (mL)	114.70 (104.00, 127.90)	107.40 (82.40, 117.10)	0.099
Clinical information			
Trace iron (μmol/L)	8.54 ± 0.47	8.74 ± 0.26	0.401
HGB (g/L)	126.38 ± 10.35	119.33 ± 8.37	0.057
HCT (%)	37.54 ± 3.37	35.85 ± 2.3	0.127
MCV (fL)	81.17 ± 3.2	82.34 ± 5.04	0.489
GDS			
Adaptive behavior	55.00 (50.00, 62.00)	—	—
Gross motor	69.66 ± 8.20	—	—
Fine motor	45.00 (36.00, 49.00)	—	—
Language	35.00 (32.00, 39.00)	—	—
Personal social behavior	50.86 ± 7.97	—	—
CARS	35.03 ± 2.95	—	—

Abbreviations: CARS: Childhood Autism Rating Scale; GDS: Gesell Developmental Schedules; GM: gray matter; HC: healthy control; HCT: Hematocrit; HGB: Hemoglobin; ICV: intracranial volume; MCV: mean corpuscular volume; MY: myelin volume; SyMRI: synthetic magnetic resonance imaging; WM: white matter.

**TABLE 4:** Comparison of characteristics and assessment scores between the mild‐to‐moderate and severe ASD subgroups.

	Mild/moderate (*N* = 18)	Severe (*N* = 11)	*p*
Gender (male/female)	15/3	8/3	0.832
Age (months)	36.00 (36.00, 48.00)	48.00 (24.00, 48.00)	0.907
Weight (kg)	15.59 ± 2.51	16.20 ± 2.90	0.618
SyMRI			
ICV (mL)	1356.78 ± 142.42	1362.18 ± 142.69	0.922
WM (mL)	362.00 (327.23, 380.72)	381.30 (369.60, 394.55)	0.256
GM (mL)	664.35 (561.07, 746.07)	723.60 (667.95, 745.15)	0.580
MY (mL)	117.04 ± 22.19	116.07 ± 16.35	0.894
Clinical information			
trace iron (μmol/L)	8.50 ± 0.49	8.69 ± 0.52	0.702
HGB (g/L)	126.86 ± 11.41	135.50 ± 6.36	0.254
HCT (%)	37.42 ± 3.83	37.75 ± 2.74	0.845
MCV (fL)	81.21 ± 3.41	81.10 ± 3.12	0.949
GDS			
Adaptive behavior	59.00 (52.25, 68.75)	50.00 (47.00, 58.50)	0.074
Gross motor	72.28 ± 6.27	65.36 ± 9.44	0.047
Fine motor	45.50 (45.00, 49.00)	35.00 (34.50, 40.50)	0.008
Language	36.00 (35.00, 39.75)	33.00 (28.50, 36.50)	0.078
Personal social behavior	53.00 ± 7.36	47.36 ± 7.99	0.072
CARS	33.50 (32.00, 34.75)	38.00 (37.00, 38.50)	0.000*

*Note*: * indicates statistically significant difference.

Abbreviations: CARS: Childhood Autism Rating Scale; GDS: Gesell Developmental Schedules; GM: gray matter; HCT: Hematocrit; HGB: Hemoglobin; ICV: intracranial volume; MCV: mean corpuscular volume; MY: myelin volume; SyMRI: synthetic magnetic resonance imaging; WM: white matter.

### 3.2 Voxel‐Wise Analysis of QSM

**FIGURE 3:**
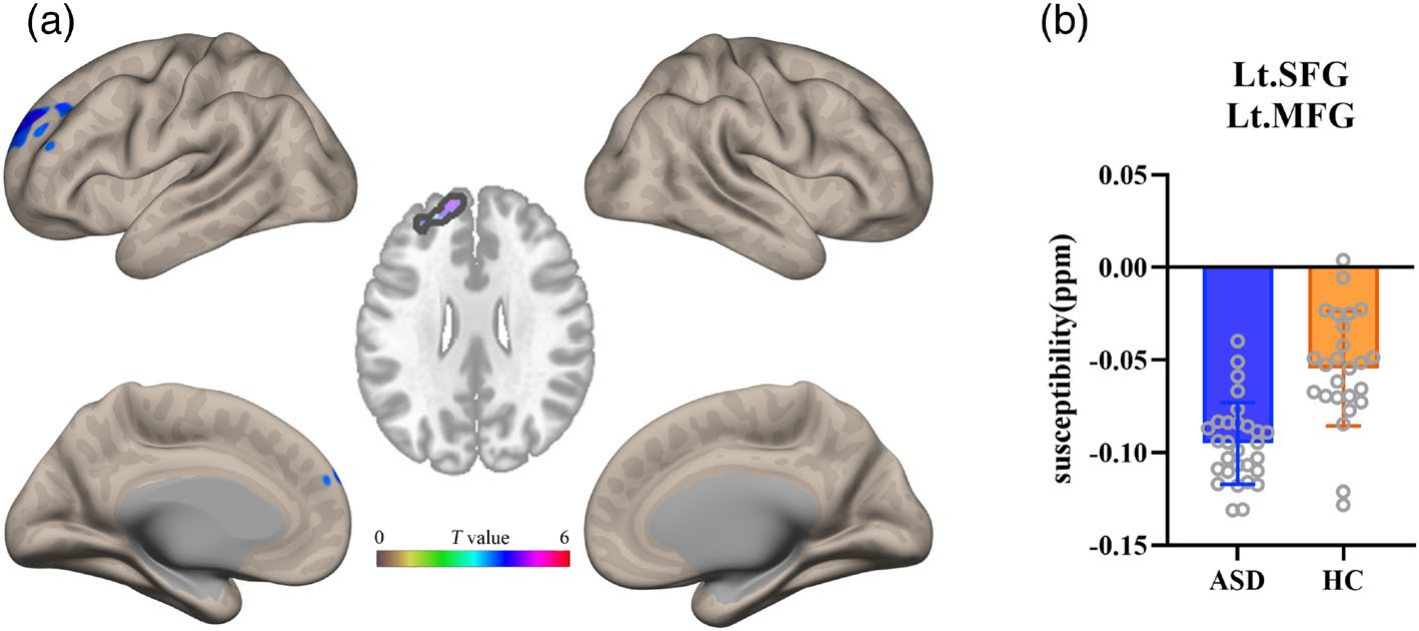
(a) Voxel‐wise QSM map comparisons between children with ASD and healthy control groups showing significant different susceptibility values, displayed in axial and surface views. (b) Group comparison of mean susceptibility value extracted from significant clusters identified in (a).

Voxel‐based analyses revealed significantly altered susceptibility values in children with ASD compared to HC (*p* < 0.05, FWE correction; Table 5, Figure 3). Specifically, children with ASD exhibited significantly lower susceptibility values in clusters located in the left superior frontal gyrus (SFG) and the left middle frontal gyrus (MFG) relative to HC participants. The mean susceptibility values extracted from these clusters also differed significantly between groups (Figure 3b, *p* < 0.05).

**TABLE 5:** Voxel‐wise comparison of QSM maps between the ASD and HC groups.

Voxel	MNI			Anatomical regions	Peak *T* value	*p*
	*X*	*Y*	*Z*			
212	−12	56	26	Lt.SFG Lt.MFG	5.55	< 0.001*

*Note*: The results were thresholded using a voxel‐level height threshold of *p* < 0.001 (uncorrected) combined with a cluster‐level threshold for multiple comparisons using family‐wise error (FWE) correction at *p* < 0.05. SFG: superior frontal gyrus; MFG: middle frontal gyrus; lt.: left. * indicates statistically significant difference.

However, voxel‐wise comparisons between the mild‐to‐moderate and severe ASD subgroups did not reveal any clusters that survived FWE correction at *p* < 0.05.

### 3.3 Voxel‐Wise Analysis of Quantitative T1, T2, PD Mappings

**FIGURE 4:**
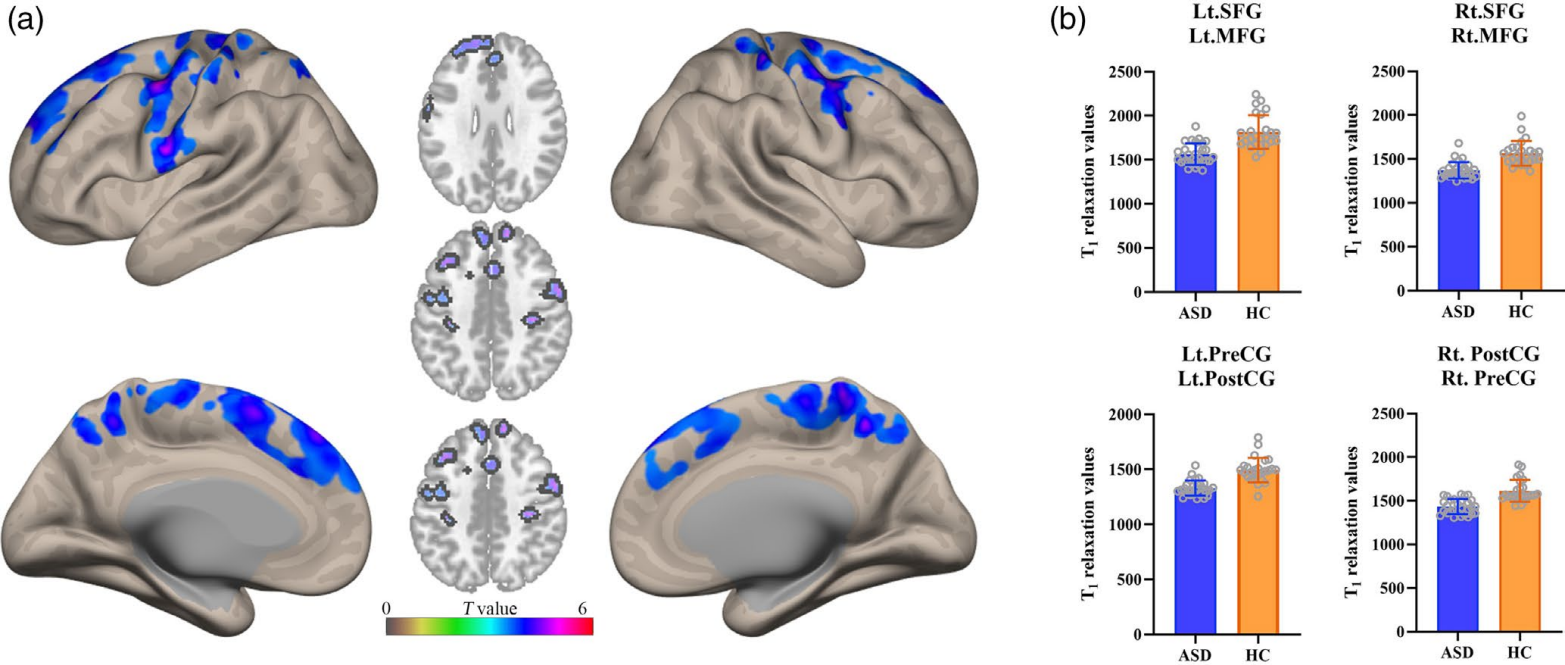
(a) Voxel‐wise T1 map comparisons between children with ASD and healthy control groups showing significant different susceptibility values, displayed in axial and surface views. (b) Group comparison of mean T1 value extracted from significant clusters identified in (a).

Voxel‐wise analysis demonstrated significantly reduced T1 relaxation values in children with ASD compared to HC (*p* < 0.05, FWE correction), with affected clusters primarily located in bilateral SFG, MFG, and precentral gyrus (PreCG) (Table 6, Figure 4).

**TABLE 6:** Voxel‐wise comparison of T1 maps between the ASD and HC groups.

Voxel	MNI					
	*X*	*Y*	*Z*	Anatomical regions	Peak *T* value	*p*
750	−6	11	62	Lt.SFG Lt.MFG	5.11	< 0.001*
501	3	−49	56	Rt.PostCG Rt.PreCG	5.50	< 0.001*
315	−48	−1	50	Lt.PreCG Lt.PostCG	5.88	< 0.001*
258	48	2	53	Rt.SFG Rt.MFG	5.29	< 0.001*

*Note*: The results were thresholded using a voxel‐level height threshold of *p* < 0.001 (uncorrected) combined with a cluster‐level threshold for multiple comparisons using family‐wise error (FWE) correction at *p* < 0.05. SFG: superior frontal gyrus; MFG: middle frontal gyrus; PostCG: postcentral gyrus; PreCG: precentral gyrus; Lt.: left; Rt.: right. * indicates statistically significant difference.

Voxel‐wise comparison between the mild‐to‐moderate and severe ASD subgroups did not reveal any significant differences in T1 relaxation values after FWE correction (*p* > 0.05). Additionally, no significant group differences were observed in T2 and PD values based on voxel‐wise analysis (*p* < 0.05, FWE correction).

### 3.4 Voxel‐Wise Analysis of Quantitative CBF Mappings

Voxel‐wise analysis revealed no significant differences in CBF maps between children with ASD and HC after FWE correction (*p* > 0.05) (Table S2). Likewise, no significant CBF differences were observed between the mild‐to‐moderate and severe ASD subgroups following FWE correction (*p* > 0.05).

### 3.5 Correlation Analysis

**FIGURE 5:**
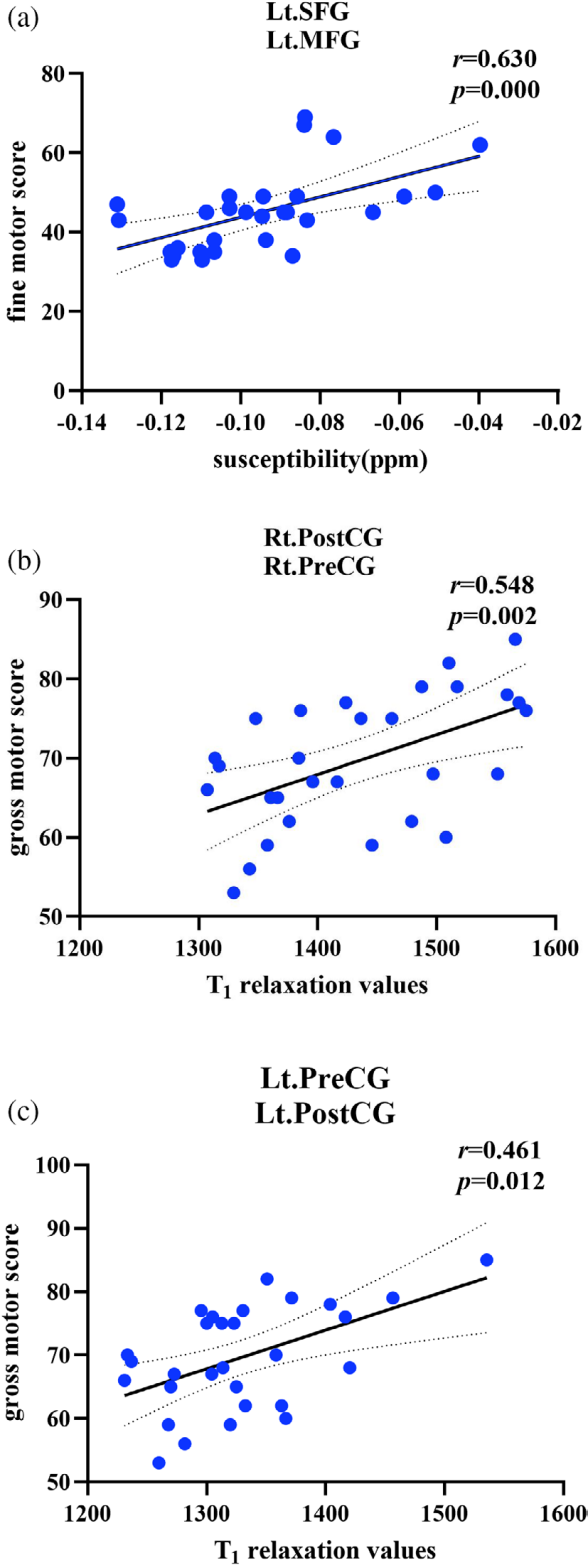
Correlation analyses between regional quantitative imaging metrics and developmental scale scores in children with ASD: (a) QSM values and fine motor scores in the left superior frontal gyrus (SFG) and left middle frontal gyrus (MFG); (b) T1 values and gross motor scores in the right postcentral gyrus (PostCG) and precentral gyrus (PreCG); (c) T1 values and gross motor scores in the left PreCG and PostCG.

Correlation analyses revealed significant associations between specific neuroimaging parameters (QSM susceptibility and T1) and developmental scores in children with ASD (Figure 5). Susceptibility values in the left SFG and left MFG were positively correlated with fine motor scores (*r* = 0.630, *p* < 0.001). T1 relaxation values in the right PreCG and PostCG showed a significant positive correlation with gross motor scores (*r* = 0.548, *p* = 0.002), and a similar positive correlation with gross motor scores was observed in the left PreCG and PostCG (*r* = 0.461, *p* = 0.012). No significant correlations were observed between the quantitative imaging values and other GSD and CARS scores (Table S3).

Additionally, no significant correlations were observed between total ICV and either susceptibility or T1 relaxation values in the identified brain regions (*p* > 0.05).

### 3.6 Diagnostic Performance of Regional Quantitative Imaging Metrics

ROC curve analysis demonstrated excellent diagnostic performance of specific regional quantitative imaging metrics in distinguishing children with ASD from HCs. Susceptibility values in the combined left SFG and left MFG yielded an AUC of 0.858, while T1 relaxation values in the same region achieved an AUC of 0.905. T1 relaxation values in the combined right PreCG and PostCG and in the combined left PreCG and PostCG yielded AUCs of 0.892 and 0.908, respectively. Additionally, T1 values in the combined right SFG and MFG achieved an AUC of 0.905.

Although combining QSM and T1 values in the left SFG and MFG reached a higher AUC of 0.937, DeLong's test showed no statistically significant improvement (*p* > 0.05) over the individual QSM or T1 measures.

**FIGURE 6:**
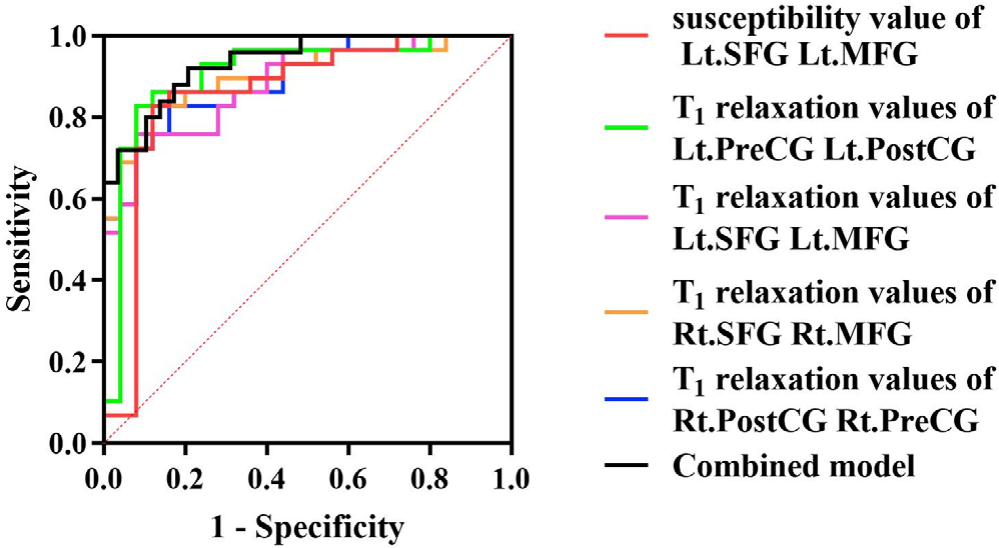
ROC curve analysis of QSM, T1 and their combined metrics across selected brain regions between the ASD and HC groups. Lt.: left; MFG: middle frontal gyrus; PostCG: postcentral gyrus; PreCG: precentral gyrus; Rt.: right; SFG: superior frontal gyrus. Combined model: Susceptibility and T1 values in left SFG and MFG.

The corresponding optimal cut‐off values, specificities, and sensitivities for regional measures and the combined model are provided in Table 7 and illustrated in Figure 6.

**TABLE 7:** Diagnostic performance based on ROC analysis.

	Cut‐off value	Specificity (%)	Sensitivity (%)	AUC (95% CI)	*p* ^a^
Susceptibility value					
Lt.SFG Lt.MFG	−0.080	0.827	0.880	0.857 (0.744, 0.972)	0.073
T1 relaxation values					
Lt.SFG Lt.MFG	1429.678	0.828	0.920	0.884 (0.823, 0.986)	0.109
Rt.PostCG Rt.PreCG	1524.176	0.758	0.840	0.892 (0.810, 0.975)	0.245
Lt.PreCG Lt.PostCG	1372.881	0.827	0.920	0.907 (0.817, 0.998)	0.501
Rt.SFG Rt.MFG	1429.678	0.827	0.920	0.904 (0.823, 0.986)	0.382
Combined model					
Lt.SFG Lt.MFG	0.248	0.793	0.920	0.936 (0.877, 0.996)	—

*Note*: SFG: superior frontal gyrus; MFG: middle frontal gyrus; PostCG: postcentral gyrus; PreCG: precentral gyrus; Lt.: left; Rt.: right. Combined model: susceptibility and T1 values in left SFG and MFG.

^a^ AUC of combined model was compared with AUC of the other values using Delong test.

### 3.7 ROI Analysis of the Basal Ganglia

Independent sample *t*‐test showed that the magnetic susceptibility value of the left caudate nucleus was lower in children with ASD compared to HCs (*p* < 0.05); however, this difference did not survive FDR correction. No significant group differences were observed in the other basal ganglia regions. All ROI analysis results are summarized in Table S1.

## 4 Discussion

This study employed multiple quantitative MRI techniques, including QSM, SyMRI, and 3D‐pCASL, to perform voxel‐based brain analysis in children with ASD. We investigated associations between spatially co‐localized brain magnetic susceptibility, relaxation parameters and cerebral perfusion with clinical behavioral scores, and further evaluated their diagnostic performance. Our results identified significantly reduced susceptibility values. Additionally, children with ASD showed decreased T1 relaxation values in bilateral SFG, MFG, PreCG, and PostCG. Quantitative measures from these regions showed significant correlations with clinical developmental scores and demonstrated strong diagnostic potential for early ASD identification.

In children, iron deficiency is strongly associated with neurodevelopmental disorders [25], while excessive iron deposition is linked to progressive neuronal degeneration [26]. Our findings of significantly reduced susceptibility in the left MFG and SFG align with a previous study reporting decreased frontal lobe iron content in children with ASD compared to typically developing controls [15]. Critically, we observed that this reduced susceptibility in the left MFG and SFG was positively correlated with lower fine motor scores. The frontal lobe is critically involved in emotion regulation and executive function [27]. Notably, the prefrontal cortex incorporates the premotor cortex [28], where electrical stimulation evokes complex coordinated movements such as whole‐hand grasping [29]. Iron is crucial for fundamental neurodevelopmental processes in these regions, including myelination, neurotransmitter synthesis (e.g., dopamine), and mitochondrial energy production [30]. Reduced iron availability may therefore impair the maturation and efficiency of neural circuits within the left MFG and SFG, specifically those involved in the precise motor planning, timing, and coordination required for fine motor skills. The observed reduction in susceptibility values within these frontal regions in ASD may compromise neural circuitry integrity, potentially underlying deficits in fine motor skill execution.

The observed iron deficiency, indicated by reduced QSM, was lateralized to the left SFG and MFG. This unilateral finding may be linked to the functional lateralization of the brain [31, 32, 33]. Given that all participants were right‐handed, the left hemisphere is typically dominant for fine motor skills and motor planning. The neural circuits within the left SFG and MFG that underpin these precise functions are likely to exhibit high levels of neural activity [34], leading to greater metabolic demand. Iron is an essential element for fundamental metabolic processes, including oxidative phosphorylation and myelin synthesis [35]. Therefore, regions with higher metabolic demands may have a greater physiological reliance on iron homeostasis. In the context of ASD, these high‐demand left frontal regions might be disproportionately vulnerable to iron deficiency. This pathophysiological model is strongly supported by the specific correlation we found between left frontal QSM and fine motor scores. The lack of a corresponding effect in the right frontal lobe suggests that the neuropathology in preschool ASD is not diffuse but rather targets specific functional networks, with those subserving dominant‐hemisphere motor functions being particularly affected.

Moreover, cerebral iron deficiency impairs myelination and oligodendrocyte dysfunction [36, 37], both essential for efficient neuronal transmission. Reduced myelin disrupts neural signaling and may underlie core ASD symptom [38, 39], a mechanism supported by postmortem findings of diminished myelin density in the prefrontal cortex of autistic individuals [40]. We also observed significantly decreased T1 values in the same frontal regions (left MFG and SFG), but the interpretation of concurrent QSM and T1 reductions requires careful consideration of their biophysical determinants. While reduced QSM in the left SFG/MFG strongly suggests local iron deficiency, the concomitant decrease in T1 is paradoxical, as iron depletion alone would be expected to prolong T1. This suggests that the T1 shortening is driven by a competing microstructural alteration, most likely involving myelin [41]. We hypothesize that in the left frontal lobe, a process of atypical or compensatory myelination is occurring [42], which dominates the T1 signal and overshadows the effect of iron deficiency.

Our voxel‐wise and supplementary ROI analyses both indicated no significant QSM differences in the basal ganglia of preschool children with ASD, contrasting with findings in some studies of older individuals with ASD [13, 14, 15]. This discrepancy may stem from several factors. First, our single‐center cohort was relatively small, predominantly male and included mostly children with mild‐to‐moderate ASD severity (mean CARS score: 35), differing from studies with broader demographic and clinical profiles. Second, by focusing on a young cohort (2–6 years), we targeted a specific developmental window that may exhibit distinct iron deposition patterns compared to older individuals.

Clinical laboratory tests of systemic iron status, including serum iron levels, hemoglobin concentration, and MCV, showed no significant differences between ASD and HC groups, nor were they correlated with brain susceptibility values. An important ancillary finding of our study is the dissociation between systemic and cerebral iron status. Despite observing regional brain iron deficiency in the ASD group using QSM, conventional peripheral blood markers of iron status (trace iron, hemoglobin, hematocrit) showed no significant group differences and were uncorrelated with brain susceptibility values. This suggests that the brain iron dysregulation in preschool ASD may be a localized phenomenon, potentially arising from altered iron transport across the blood–brain barrier or aberrant regional iron utilization within the brain, rather than a reflection of systemic iron deficiency.

T1 relaxation time is influenced by multiple tissue properties, including myelin content, iron concentration, and water content [43, 44]. The observed reductions in T1 relaxation time in the bilateral frontal and sensorimotor cortices could arise from several microstructural alterations beyond, or in conjunction with, iron content. Firstly, myelin content is a primary driver of T1 contrast [45, 46]. While some ASD studies report myelination deficits, others, including in related neurodevelopmental conditions have observed elevations in myelin volume which could shorten T1 [47, 48]. Secondly, T1 is sensitive to axonal density and organization. Increased axonal packing or more coherent fiber alignment can reduce T1 independently of myelin [49, 50]. Thirdly, a decrease in tissue water content associated with more mature or densely packed neuropil would also lead to T1 shortening [44]. Finally, our findings are consistent with reports of reduced gray‐white matter boundary contrast in the SFG of individuals with ASD [51, 52], which is thought to reflect atypical intracortical myelination patterns. The T1 reductions we observe might thus represent a complex, and potentially region‐specific, interplay of these factors. T1 relaxation values in the bilateral PreCG and PostCG were significantly correlated with gross motor scores. The PreCG and PostCG, forming the core of the sensorimotor cortex, are anatomically adjacent and functionally tightly coupled. Altered tissue properties (indicated by decreased T1 values) within this bilateral sensorimotor network may reflect microstructural abnormalities that disrupt sensorimotor integration, thereby contributing to impaired gross motor coordination in children with ASD. This interpretation is supported by evidence that certain antidepressant medications ameliorate core ASD symptoms, including social deficits and motor impairments [53]. These findings suggest pathological alterations in the somatosensory cortex may represent a pathophysiological substrate underlying gross motor abnormalities in ASD.

It is noteworthy that we did not find significant microstructural differences between the mild‐to‐moderate and severe ASD subgroups. This is likely attributable to the limited statistical power of our study, as the severe subgroup contained only 11 participants, reducing our ability to detect effects of smaller magnitude.

This study found no significant alterations in CBF, T2 relaxation times, or PD values in the examined brain regions of children with ASD compared to HCs. As T2 relaxation time is primarily influenced by tissue properties such as neuronal injury, edema, inflammation, and iron loading [17, 54, 55], the absence of significant T2 changes in our ASD cohort may suggest that acute pathological processes, such as cerebral perfusion deficits, significant edema, or overt inflammation, were not characteristic features in this population. Alternatively, compensatory mechanisms related to cerebral perfusion or other neurophysiological factors may mitigate observable differences, resulting in comparable T2, PD and CBF measurements between groups [56]. The use of dexmedetomidine, while necessary for successful scan acquisition in this young age group and applied consistently across all participants, is known to reduce global CBF. This universal suppression may have compressed the dynamic range of our CBF measurements, potentially obscuring subtler, region‐specific perfusion alterations associated with ASD.

Evaluation of diagnostic performance revealed that both individual and combined regional quantitative metrics effectively differentiated children with ASD from controls. The highest nominal accuracy was achieved by integrating QSM and T1 measures. The lack of a statistically significant enhancement over the top‐performing single metric may be attributable to the high collinearity between these microstructural parameters or the limited sample size for this specific sub‐analysis. Nevertheless, the strong performance across multi‐parameter underscores the general utility of quantitative MRI biomarkers.

The high diagnostic accuracy of both QSM and T1 metrics in distinguishing preschool children with ASD from HCs is noteworthy. This performance is comparable to that of other MRI‐derived biomarkers reported in ASD studies, and even superior to these biomarkers in some regions, such as cortical thickness, surface area, or functional connectivity measurements [57, 58, 59], which typically show AUCs in the range of 0.620–0.850. This supports the potential clinical utility of T1‐ and QSM‐based multi‐parametric MRI as sensitive imaging markers for early neurodevelopmental assessment.

## 5 Limitations

First, all MRI scans were acquired on a single MRI scanner to minimize technical variability. While this enhances the internal consistency of protocols, it may limit the generalizability of findings across different imaging platforms. Second, participant recruitment was challenging due to the need for sedation in young children. Future longitudinal studies with larger and more diverse cohorts are needed. Third, the relatively broad age range (2–6 years) spans critical developmental stages with varying myelination trajectories, potentially introducing age‐related confounding effects. Fourth, although age‐specific CHN‐PD tissue priors were applied for spatial normalization, residual registration errors may persist, particularly in the youngest participants with rapidly evolving neuroanatomy. Fifth, while QSM predominantly reflects iron content, contributions from other paramagnetic substances (e.g., copper, zinc) cannot be entirely excluded [60]. Our interpretation assumes that iron content is the primary driver of both magnetic susceptibility and relaxometry changes (via myelination), though alternative biomolecular contributors (e.g., proteins, lipids) should be systematically explored in future mechanistic studies. Sixth, while we integrated several quantitative technologies, we did not include diffusion MRI. Future studies incorporating diffusion tensor or diffusion kurtosis imaging would provide valuable insights into axonal microstructure and WM organization, enabling a more comprehensive characterization of brain microstructural alterations in preschool ASD. Furthermore, an isotropic smoothing kernel (8 mm FWHM) used in our study is commonly used in whole‐brain voxel‐wise analyses to balance signal‐to‐noise ratio and anatomical variability across subjects; however, this relatively large kernel may have reduced the sensitivity to detect fine‐grained microstructural differences, particularly in thin cortical regions. Finally, the quantitative maps were derived from sequences with differing spatial resolutions. We did not apply partial volume correction, which might influence the quantitative values, particularly at tissue boundaries. Future studies would benefit from incorporating such corrections to enhance the accuracy of the measurements.

## 6 Conclusion

This quantitative MRI study reveals converging and complementary microstructural alterations in the frontal lobes and sensorimotor cortices (precentral/postcentral gyri) of preschool‐aged children with ASD, as assessed through susceptibility and relaxation time measures. Specifically, our findings demonstrate reduced iron content in prefrontal regions, and atypical myelination patterns across identified neural circuits. The use of multimodal MRI techniques not only deepens our understanding of neurodevelopmental abnormalities in ASD, but also highlights the association between imaging biomarkers and clinical evaluation. These findings underscore the potential of such imaging biomarkers to support early ASD diagnosis and guide targeted intervention strategies.

## Author Contributions

**Changhao Wang:** methodology, writing – original draft, project administration. **Meiying Cheng and Xin Zhao:** funding acquisition, writing – review and editing. **Meiying Cheng, Yu Lu, and Jinxia Guo:** data curation, formal analysis. **Xueyan Liu:** visualization. **Xin Zhao:** writing – review and editing, validation. **Changhao Wang, Zhanqi Feng, and Shipeng Liu:** software, investigation.

## Funding

This research was supported in part by the National Natural Science Foundation of China (no. 82472046) and Henan Province Science and Technology Research (no. 232102311091).

## Ethics Statement

This study conformed to the Declaration of Helsinki on Human Research Ethics standards and received approval from the Third Affiliated Hospital ethics committee of Zhengzhou University (2024‐105‐01).

## Conflicts of Interest

The authors declare no conflicts of interest.

## Supporting information


**Table S1:** Comparison of magnetic susceptibility values within region of interest in children with ASD.
**Table S2:** Voxel‐wise comparison of CBF maps between the ASD and HC groups.
**Table S3:** Correlation analysis between the ASD and HC groups.
