# Supplementary material for: Evaluation of Brain Microstructural Alterations in Preschool Autism Spectrum Disorder: A Voxel‐Wise Multimodal MRI Study
Source: J Magn Reson Imaging. 2026 Jan 16;63(4):1053–66. doi: 10.1002/jmri.70185 (PMC12963812; doi:10.1002/jmri.70185)
Supplement: Supplementary file 1 — Table S1: Comparison of magnetic susceptibility values within region of interest in children with ASD. Table S2: Voxel‐wise comparison of CBF maps between the ASD and HC groups. Table S3: Correlation analysis between the ASD and HC groups. [file JMRI-63-1053-s001.docx]

**Supplementary materials**

**Supplementary Table 1** Comparison of magnetic susceptibility values within region of interest in children with ASD

| ROI | ASD(N=29) | HC(N=25) | *t/Z* value | *p* value | *q* (FDR) value | *Cohen's d/r* |
| --- | --- | --- | --- | --- | --- | --- |
| Lt.Caudate nucleus^1^ | -33.18±51.73 | -6.76±43.03 | -2.02021 | 0.0485 | 0.6794 | -0.551 |
| Rt.Caudate nucleus^1^ | -17.47±45.57 | -24.27±37.61 | 0.591503 | 0.5567 | 0.7924 | 0.161 |
| Lt.Putamen^1^ | -29.81±50.93 | -36.64±49.78 | 0.496489 | 0.6216 | 0.7924 | 0.135 |
| Rt.Putamen^1^ | -29.53±40.46 | -24.72±43.30 | -0.42158 | 0.6751 | 0.7924 | -0.115 |
| Lt.Pallidum^1^ | -23.17±96.11 | -45.61±79.42 | 0.926173 | 0.3586 | 0.7924 | 0.253 |
| Rt.Pallidum^2^ | 3.14(-53.90,14.07) | -42.01(-68.58,-1.11) | -1.38352 | 0.1665 | 0.777 | 0.132 |
| Lt.Thalamus^2^ | 17.26(-72.35,98.77) | 11.71(-90.96,116.40) | -0.0688 | 0.9451 | 0.9451 | 0.218 |
| Rt.Thalamus^1^ | -36.84±111.83 | -49.34±108.03 | 0.41586 | 0.6792 | 0.7924 | 0.113 |

q(FDR) vales are False Discovery Rate adjusted *p* vales.1: Data are normally distributed, compared using independent samples *t-test*, with effect size expressed as Cohen's d.2: Data are non-normally distributed, compared using the *Mann-Whitney U* test. HC: healthy control Lt.: left;Rt.: right. * indicates statistically significant difference.

**Supplementary Table 2** Voxel-wise comparison of CBF maps between the ASD and HC groups

| Voxel | MNI | | | Anatomical Regions | Peak T value | *p* uncorrected value | *p* FWE value |
| --- | --- | --- | --- | --- | --- | --- | --- |
|  | X | Y | Z |  |  |  |  |
| 523 | -33 | -76 | 17 | Lt. MOG | 3.16 | 0.105 | 0.381 |
| 109 | -48 | -55 | 41 | Lt. IPL | 3.10 | 0.453 | 0.808 |
| 179 | -3 | 32 | -16 | Lt. REC | 3.00 | 0.332 | 0.701 |

The results were thresholded using a voxel-level height threshold of *p* <0.005 (uncorrected) combined with a cluster-level threshold for multiple comparisons using family-wise error (FWE) correction at *p* < 0.05. MOG: Middle Occipital Gyrus; IPL: Inferior Parietal Lobule; REC: Rectus Gyrus; Lt.: left；Rt.: right. * indicates statistically significant difference.

**Supplementary Table 3** Correlation analysis between the ASD and HC groups

|  |  | Adaptive Behavior | Gross Motor | Fine Motor | Language | Personal-Social Behavior | CARS |
| --- | --- | --- | --- | --- | --- | --- | --- |
| Susceptibility value |  |  |  |  |  |  |  |
| Lt.SFG  Lt.MFG | *r* value  *p* value | 0.220  0.252 | 0.321  0.090 | 0.630  <0.001* | 0.336  0.075 | 0.279  0.142 | -0.140  0.468 |
| T1 relaxation values |  |  |  |  |  |  |  |
| Lt.SFG  Lt.MFG | *r* value  *p* value | -0.140  0.470 | 0.389  0.037 | -0.082  0.674 | -0.083  0.669 | -0.026  0.892 | 0.113  0.558 |
| Rt.PostCG  Rt.PreCG | *r* value  *p* value | 0.041  0.835 | 0.548  0.002* | 0.139  0.471 | 0.172  0.373 | 0.099  0.609 | -0.065  0.736 |
| Lt.PreCG  Lt.PostCG | *r* value  *p* value | -0.153  0.427 | 0.461  0.012* | -0.098  0.613 | 0.009  0.962 | -0.048  0.804 | 0.162  0.401 |
| Rt.SFG  Rt.MFG | *r* value  *p* value | 0.024  0.900 | 0.396  0.033 | -0.133  0.493 | 0.019  0.922 | -0.024  0.903 | 0.021  0.915 |

MOG: Middle Occipital Gyrus; IPL: Inferior Parietal Lobule; REC: Rectus Gyrus; Lt.: left；Rt.: right. * Significant correlations are indicated in bold and remained significant after Bonferroni correction for multiple comparisons (corrected significance threshold: *p* < 0.05 / ROIs ). CARS: Childhood Autism Rating Scale.
